# Creating the sustainable conditions for knowledge information sharing in virtual community

**DOI:** 10.1186/s40064-016-2702-7

**Published:** 2016-07-08

**Authors:** Jiangtao Wang, Jianmei Yang, Quan Chen, Sang-Bing Tsai

**Affiliations:** Zhongshan Institute, University of Electronic Science and Technology of China, No. 1, Xueyuan Road, Zhongshan, Guangdong 528400 China; School of Business Administration, South China University of Technology, Guangzhou, 510641 China; School of Economics & Management, Shanghai Maritime University, Shanghai, 201306 China; Law School, Nankai University, Tianjin, 300071 China; China Academy of Corporate Governance, Nankai University, Tianjin, 300071 China; School of Business, Dalian University of Technology, Panjin, 124221 China

**Keywords:** Web community, Knowledge sharing, Web management, Service management

## Abstract

**Introduction:**

Encyclopedias are not a new platform for the distribution of knowledge, but they have recently drawn a great deal of attention in their online iteration. Peer production in particular has emerged as a new mode of providing information with value and offering competitive advantage in information production.

**Case description:**

Large numbers of volunteers actively share their knowledge by continuously editing articles in Baidu encyclopedias. Most articles in the online communities are the cumulative and integrated products of the contributions of many coauthors.

**Discussion and Evaluation:**

Email-based surveys and objective data mining were here used to collect analytical data. Critical mass theory is here used to analyze the characteristics of these collective actions and to explain the emergence and sustainability of these actions in the Baidu Encyclopedia communities.

**Conclusions:**

These results show that, based on the collective action framework, the contributors group satisfied the two key characteristics that ensure the collective action of knowledge contribution will both take place and become self-sustaining. This analysis not only facilitates the identification of collective actions related to individuals sharing knowledge in virtual communities, but also can provide an insight for other similar virtual communities’ management and development.

## Background

Peer production is a new mode of production that has recently emerged from software and content production. This mode, which is based on sharing and cooperation, has spawned almost all of the mature operating system such as GNU/Linux and various BSD systems and innumerable other free software applications. The advent of so-called Web 2.0 technology has infused this mode with new vigor. One of the typical cases of peer production based on Web 2.0 technology is the Online Encyclopedia.

With the development of Web 2.0, Internet users ceased to be solely passive recipients of information. They can now share their own knowledge and interact with others in collaborative workspaces (Dave and Koskela 2009). Net users can freely create and edit web page content using any Web browser. The Wiki system is a new technology that can be used to establish this collaborative workspace. Wiki technology has been demonstrated to be an unconventional technology with high potential for affecting knowledge creation, sharing, integration, and utilization (Ashton 2010; Cho et al. 2010).

The Online Encyclopedia is based on the Wiki system. It allows the creation and editing of encyclopedia articles by anyone who wishes to contribute. The primary purpose of online encyclopedias is to provide free access to knowledge representing the editors’ consensus on the subject of each article. Individuals generally engage by contributing what they know to relevant entries. The Online Encyclopedia has accelerated the rise of knowledge sharing. Individual participation can be considered knowledge sharing behavior by taking the content of online encyclopedias into account. The wiki system has also been used for knowledge management by many businesses as an affordable and effective Intranet.

Generally, a virtual community can be viewed as cyberspace supported by computer-based information technology centered upon communication and interaction of participations to generate member-driven contents (Lee et al. 2003). Online encyclopedias such as Wikipedia, Baidu Encyclopedia and Wikia all have these features. The process of collecting and sharing knowledge is the collective action of large numbers of co-authors in these communities. The latest versions of online encyclopedias articles are general the collective product of a large numbers of individual contributions. Interestingly, all the participations are volunteers. Those who create content do not receive any material benefits. Anyone with Internet access also can search and browse Wikipedia or the Baidu-Encyclopedia entries for free. These properties provide online encyclopedia content as a public good (Rahman 2009). Intuitively, one would think that rational individuals would not regularly contribute content to these self-organized communities. In truth, there is a free-ride element to any collective action. Generally, there are two major challenges to these voluntary collective actions on these virtual communities: the start-up problem and discontinuity problem. Wikipedia and Baidu Encyclopedia have both survived the start-up problem. Wikipedia is the best-known and largest online encyclopedia. It has achieved sustainability and shown rapid and continual growth. The Baidu Encyclopedia is one of the largest Chinese online encyclopedias. Although it started later than Wikipedia, it can make better use of its advantages as later starts and develop quickly in recent years. These successful online communities provide a fruitful basis for understanding the social mechanisms, and the attributes that promote successful collective action of voluntary knowledge collection and sharing (Prasarnphanich and Wagner 2008).

However, most existing literature on online encyclopedias focused on Wikipedia. Some literature focused on investigating the user’s motivation in Wikipedia (Hendriks 1999; Yang and Lai 2010). For example, Yang and Lai (2010) investigated the motivations of Wikipedia content contributors. Some literature investigated the relationships between users on Wikipedia (Jankowski-Lorek et al. 2013; Ermann et al. 2013; Li et al. 2012; Silva et al. 2011). For example, Silva et al. (2011) investigated the relationships within and between category networks in Wikipedia. Some literature studied the contributors’ behavior (Zhao et al. 2013; Iba et al. 2010; Cho et al. 2010). For example, Zhao et al. (2013) investigated whether these user interests and resources can increase contribution value for different types of users in Wikipedia, and Iba et al. (2010) analyzed the editing patterns of Wikipedia contributors.

Unlike the existing literature on the online encyclopedia, critical mass theory originated in physics and has been used in social sciences provides a complex theoretical model of the production of collective actions. Hence, critical mass theory is used to analyze the collective actions of knowledge sharing within online encyclopedia communities.

### Relevant theory

The large group problem in collective action has a very simple logic: the greater the number of people that is required to produce a collective good, the less the value of any single individual’s contribution. Olson (1965) then famously argued that this logic implies that, barring additional incentives, no individual will be rational to contribute, and voluntary collective action in large group is doom to failure. However, we can empirically observe many instances of mass actions. Oliver et al. (1985) then presented the *Critical Mass Theory* in response to Olson’s famous large group argument logic. Marwell et al. (1988), Oliver and Marwell (1988), Marwell and Oliver (1993) later improved the theory further. Markus (1987) ever applied this theory to explain the diffusion of interactive media. Critical mass theory mainly concerns the collective actions that produce a “collective good or public good,” and seeks to predict the probability, extent, and effectiveness of community action in the pursuit of the collective good (Marwell and Oliver 1993). This theory has played a major role in the development of collective theory and has provided an essential foundation for incorporating rational choice theory into the mainstay of sociological approaches to collective action (Oliver and Marwell 2001; Centola 2013). Specifically, Olson (1965) argued that the only way to initiate sizable collective action is to add “selective incentives,” such as punishments for defection or reward for cooperation, Marwell and Oliver (1993) showed that contributions to collective action can create “positive externalities,” whereby initial contributions create more incentive for subsequence actors. So, one issue with critical mass theory is to address the condition under which collective action can emerge and become self-sustaining. According to this theory, the likelihood of successfully facilitating the collective good depends primarily on two crucial factors, one of which takes the form of the production functions concerning the contribution of resources, and another one of which is the development of group heterogeneity. These can be elaborated upon as follows:

Production functions describe the relationship between individuals’ contribution of resources and the achievement of the public good. Different types of production functions create dramatically different dynamics in otherwise similar situations and so produce different outcomes. Generally, decelerating production function (i.e. decelerating marginal returns) involves decreasing marginal returns to contributions and refers to situations in which the first few units of resources contributed have the largest effect on the collective good and subsequent contributions progressively less. In contrast, accelerating production function (i.e. accelerating marginal returns) involves increasing marginal returns and causes subsequent contributions to generate progressively larger payoffs. In this way, each contribution makes the next one more likely. Noticeably, the collective good with accelerating production function will suffer the daunting start-up problems but optimization and sustainability can be achieved once the initial contributions are obtained.

Group heterogeneity is favorable to collective action. The significance of this factor has been widely recognized since the emergence of critical mass theory. As Oliver et al. (1985) pointed out, the heterogeneity of the population—specifically, i.e., the number of deviants and the magnitude of their deviance—is one key to predicting the likelihood, extent and effectiveness of collective action. The main focus on heterogeneity in this work concerns the interest of and resources provided by the communities that edit these two encyclopedias. Interest here refers to the motivations that drive individuals to contribute and resources refers to the time, energy, talent or other contribution that an individual can make or depend on to further the public good. Only when the interests of the group are heterogeneous will the number of highly interested or highly resourceful reach critical mass. This will happen even when the mean interest or resource level is low. The likelihood of successful collective action depends primarily on reaching critical mass of individuals who make significant contributions to the project, even if the majority of participants do little or nothing.

### Baidu Encyclopedia community

Undoubtedly, Wikipedia is the most extensive and well-known Wiki application. Wikipedia has also attracted growing academic attention due to its popularity and unconventional modes of operation (Ashton 2010; Cho et al. 2010; Rahman 2009; Prasarnphanich and Wagner 2008; Royal and Kapila 2009). Baidu Encyclopedia, like Wikipedia, is also the typical of wiki applications, and it is the largest online encyclopedia in China. Test versions of the Baidu Encyclopedia were released on April 20, 2006, and, within 3 weeks, the encyclopedia had grown to more than 90,000 articles, surpassing the Chinese Wikipedia. As of October 2013, the Baidu Encyclopedia has more than 6.2 million articles, which is more than the English Wikipedia (http://baike.baidu.com/). Apparently, the Baidu Encyclopedia can make use of the advantages of its position as a late starter.

However, Baidu Encyclopedia is more like a combination of a self-organized community and other types of organizations, while the Wikipedia community has been viewed as a self-organized community. Wikipedia is a non-profit online encyclopedia, and it provides people with the freedom to share knowledge (Akiyoshi 2008). Although the Wikipedia community has a clear power structure that gives volunteer administrators the authority to exercise editorial control (Hafner 2006; Corner 2006), these administrators at least officially do not enjoy any special privileges with respect to decision-making. Instead, they are mostly limited to making edits that have project-wide effects and thus are disallowed to ordinary editors’, and to block users who make disruptive edits (https://en.wikipedia.org/wiki/ Wikipedia:Administration, Retrieved July 12, 2009). The Baidu Encyclopedia is a Chinese-language collaborative Web-based encyclopedia provided for use with the Chinese search engine Baidu (*The Baidu Story*. Baidu. Retrieved on June 3, 2011.). The Baidu Company applies a Wiki philosophy to the encyclopedia, which is mean to complement the search engine and two other services (Zhidao and Post). All articles written and edited by registered users must be reviewed by behind-the-scenes administrators before publication in order to be self-censored in accordance with the government regulations. However, the Baidu Encyclopedia is an open-Internet encyclopedia. In this capacity, it espouses equality, collaboration, and sharing, and it provides a voluntary environment that allows collaborative contributors and maintenance in a simple and effective manner. Most low-level positions open to all volunteers, while only individual high-level administrative positions are given out by appointment. Any Internet user in the Baidu Encyclopedia can easily modify any article or create a new one.

At present, the available languages in the Baidu Encyclopedia include its primary language, simplified Chinese, as well as traditional Chinese, Korean, Vietnamese, and Japanese, which are automatically translated into simplified Chinese. All registered users’ contributions are evaluated and rewarded using a credit point system. There are two forms of assessment credits in the Baidu Encyclopedia: experience value and wealth value. Experience scores are based on what someone does, regardless of the results, and wealth scores are based primarily on results. Experience scores also relate to the level and honor within the community. Each Baidu Encyclopedia user can create a personal space in which he or she can share historical contribution information, personal characteristic information, and the honors and rewards offered by Baidu Encyclopedia. In this way, the Baidu Encyclopedia can provide large number of objective analytical data that can be used to analyze the collective knowledge sharing actions within this virtual community.

### Research method and results

The study was reviewed and approved by an institutional review board Zhongshan Institute, University of Electronic Science and Technology of China. (ethics committee)

Critical mass theory provides a complex theoretical model of the production of collective action and it can be used to explain the emergence and sustainability of collective action. There are three research questions that must be answered when using critical mass theory to examine the emergence and predict the sustainability of collective action concerning knowledge sharing in virtual communities:

1. Are the contributors’ interests heterogeneous?
2. Are these contributors’ ability to provide resources heterogeneous?
3. What is the type of production function?

As described above, the Baidu Encyclopedia and Wikipedia have both survived the start-up problem and currently provide a fruitful crop of analytical data that can be used to answer the research questions.

Generally, most editing projects in Baidu Encyclopedia are based on common areas of knowledge or interest such as hobbies, industries, popular culture, and geographic areas. Noticeably, knowledge sharing on the encyclopedia takes time and effort, and contributors rarely derive explicit material benefits other than virtual rewards and prestige among their peers. Many prior studies have preferred to use motivational theory to interpret individual knowledge-sharing behavior and confirm that motivation has a key role in the intent and behavior of knowledge sharing (Bock et al. 2005; Cabrera et al. 2006). In this way, individual interest can be measured by the motivation in collective action of knowledge sharing and the heterogeneity of interest can be indicated by the diversity of motivation.

Individual resources include the skill, energy, time, and spirit with which we are personally endowed (Oliver et al. 1985). Individual resources can also be related to individual educational attainment and career. The current work focuses primarily on the heterogeneity of these two factors.

Production function mainly refers to the relationship between achievement of public good and individuals’ contribution of resource. The type of production function is given in one of two ways: scoring rule and fitted curve.

Two methods were used to answer these questions and collect analytical data: (1) An email-based survey was performed to assess motivation; and (2) data mining was used to mine available objective data in specific sub-communities to assess individuals’ educational attainment and career distribution.

#### Interest heterogeneity

Most previous studies have shown that motivation underlying knowledge sharing comes primarily from a desire for personal growth, operational autonomy (Tampoe 1993), a sense of belonging, self-esteem, self-actualization (Stott and Walker 1995), sense of accomplishment, recognition, challenge, responsibility, and opportunity for promotion (Hendriks 1999). Yang and Lai (2010) also pointed out that motivation based on self-concept-based has a greater influence on individual knowledge-sharing behavior on Wikipedia than other motives do and that internal self-concept-based motivation is the most important motive for sharing knowledge. Self-concept-based motivations can be viewed as the generalized individual interest in collective action of knowledge sharing.

To assess the motivation of participants in Baidu Encyclopedia, we have to contact large numbers of contributors to carry out the survey. Fortunately, Baidu Encyclopedia allows a register member to contact the contributors in a form of private letter within the platform. However, many contributors would not reply the private letters. We randomly and continuously sent lots of private letters to explain our motivation of the survey. Finally, we received two hundred contributors’ replies. Hence, two hundred contributors were randomly chosen and an online survey questionnaire was conducted through interactive media, such as QQ, e-mail. The items used to assess motivation were mainly adapted from previous studied (Yang and Lai 2010) and modified for use in knowledge sharing context within Baidu Encyclopedia. Finally, 129 valid questionnaires were received after 1 week. Here, 23.3 % of participants were female, and 76.7 % were male. The average age of the respondents was 22.5 years. The motivation of the participants and their implications for heterogeneity of interest are analyzed as follows (Table 1):

**Table 1:** Statistic table of interest/motivation for participation in Baidu Encyclopedia

Interest categories/motivations	Number of participants	Rate (%)	Questionnaire items
Enjoying	99	77	I Enjoy Sharing My Knowledge with Others/Sharing My Knowledge with Others Gives Me Pleasure
Altruism/Pro-Social Behavior	98	76	Sharing My Knowledge Can Help Someone
Reciprocity	92	71	Exchanging Opinions on Specific Topics is Good Sharing Knowledge Can Promote Mutual Learning
Recognition	83	64	I Like Knowing Whether Others Approve of My Behavior/Sharing Knowledge Gives Me a Sense of Personal Achievement
Enhancing Own Learning and Insights	76	59	Sharing Knowledge Can Improve My Professional Status and Enhance My Own Learning and Insights
Future Growth of Community	75	58	Individual Sharing of Knowledge Can Help the Community Continue to Grow and Evolve
Score	46	36	Sharing Knowledge Can Earn Me More Credit
Personally-Valued Goals	32	25	Sharing Knowledge Can Improve My Image (for Example, Honors) I Like Earning Respect by Participating
Others	6	5	Others
Max	99	77	

As shown here, motivation usually involved pluralism. Notable motives included enjoyment, altruism and reciprocity. Recognition, desire to learn and desire to facilitate community growth also had great influence on individual knowledge-sharing behavior. It should be noted that earning more points was also one motivation for sharing knowledge in Baidu Encyclopedia.

#### Resource heterogeneity

**Fig. 1:**
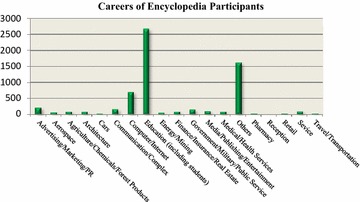
User’s career distribution

**Fig. 2:**
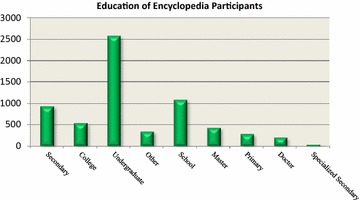
User’s educational attainment distribution

Baidu Encyclopedia allows all contributors to create a personal characteristic space, where their personal information can be mined. People with different careers have different amounts of time available and edit articles with different perspectives. In this way, career and educational attainments of users in the “physics” and the “mathematics” professional classes can be mined. Resource heterogeneity can be reflected on the distribution of these two factors as follows in Figs. 1 and 2.

Participants covered a wide range of careers. The largest group fell into the educational category, including students. Education is a very broad concept. These highly educated users may come from different disciplines and have different skills, energy and time. Intuitively, a participant in the educational category should undertake the dissemination of knowledge, especially in volunteer settings.

At the same time, the educational attainment distribution as follows.

Although online encyclopedias allow all Internet users to edit their articles, most of these whose contributors are those who have received a large amount of education. Only someone who has knowledge can share it with others. Since heterogeneity is the conditions that the collective action of knowledge contribution started and become self-sustaining. Hence, communities in which knowledge is shared should encourage more heterogeneous and well-educated people to participate.

#### Production function

Baidu Encyclopedia rewards all users’ contributions with points. The payoff of individual contributions can be measured in reward scores. The scoring rules of Baidu Encyclopedia are as follows: users can obtain three reward points by creating a new entry; user can obtain one reward point by performing simple edits to existing entries, including both additions and amendments, but they can obtain five reward additional points for complex edits, such as large improvements to content and other increases to the quantity of information offered. Once the edited entry is assessed as primary quality entry, all contributors involved obtain five reward points. After editing by subsequent contributors, the primary quality entry is again assessed as a secondary quality and senior quality entry, and then all contributors involved again obtain ten reward points and thirty reward points. Hence, score rules have shown that production function is more like an accelerating function in Baidu Encyclopedia. Monge et al. (1998) ever argued that contributions that update and upgrade collective information cause the production function to accelerate.

In order to assess the production function with quantitative method, data mining methods were used to mine the available objective data.

In Baidu Encyclopedia, all entries are sorted into different classes. At the bottom of homepage (http://baike.baidu.com/), the category navigation system can help searches for different categories. As of June 18, 2013, there were 6,235,088 articles produced by 3,249,307 contributors. Data regarding the revision history of articles in the “physics” class and the “mathematics” class were here mined. The mined data are given as “Article-Edited Time-User.” 28,267 available revision history data are mined, including 13,144 available revision history data in the “mathematics” class and 15,483 available revision history data in the “physics” class (Table 2).

**Table 2:** Article revision history

Class	Available articles	Entries	Users	Earliest time	Latest time
Mathematics	13,144	500	8013	2006-4-5 20:58	2013-6-5 8:40
Physics	15,483	500	8590	2006-4-5 19:30	2013-6-15 17:04

“Available data” means the available revision history data. “Entries” means the number of articles involving in this class. “Users” means the number of contributors in this class

The two sub-classes were integrated into a larger subclass and all users’ participation information (experience value and wealth value) was collected from their personal space.

Prasarnphanich and Wagner (2008) ever examined the number of articles in Wikipedia over time to assess content production function. Here, the primary data were sorted by time and divided into 29 time segments based on the time series (by quarter). Then the number of edits made to the articles and contributions made over time (From the earliest time to the latest time) in the sub-class are analyzed as follows.

**Fig. 3:**
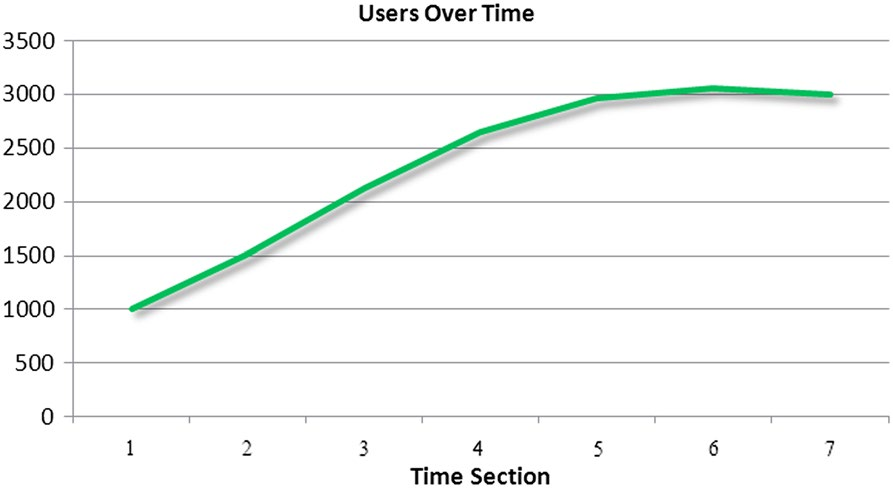
The numbers of user over time in Baidu Encyclopedia

**Fig. 4:**
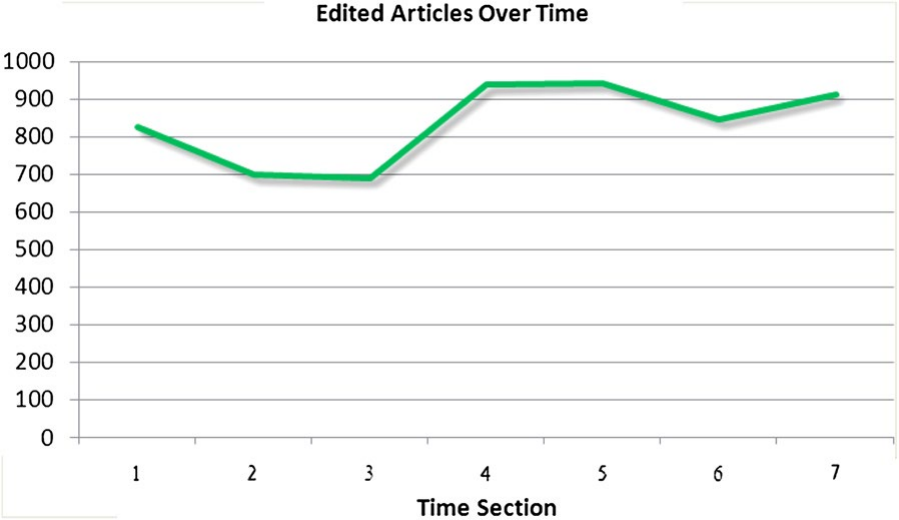
The numbers of edited articles over time in Baidu Encyclopedia

Figure 3 shows that more users have taken part in the community over time. Figure 4 shows that the number of articles edited has fluctuated over time. Figure 3 shows that more new contributors have taken part in the editing process. However, the numbers of edited articles fluctuated over time, the production function curve appears to be an accelerating function, which differs from the description with reward score system. Hence, these two figures cannot prove any consistent conclusion with respect to assessments of the type of production function. The central issue with peer production is the participation of large numbers of users. Participation here refers not only to the creation of articles but also to edits made to articles. All articles in the community are the cumulative accomplishment of all users’ contribution. In this way, there are serious limitations to using the number of articles and number of contributors to assess the type of production function.

**Fig. 5:**
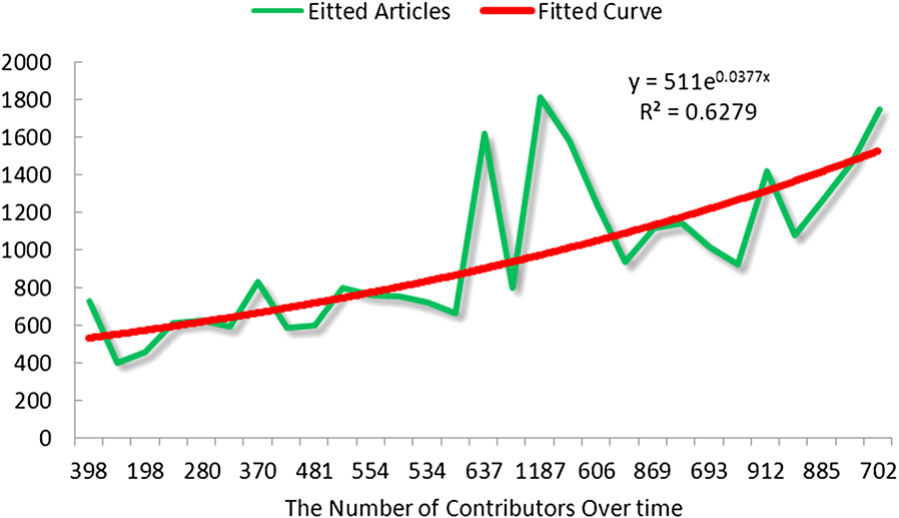
production function in sub-community

Since the quality of most is indicated by the number of edits made (Lih 2004). Here, the number of contributors versus the number of edits over time can be used to assess the production function. The fitted results are as follows (Fig. 5):

Although the actual scatter fluctuated wildly over time, the fitted curve indicates a pattern of accelerating production function, which coincides with the description in the reward score system. This accelerating production function can draw the interest of subsequent contributors, which can draw even more subsequent contributors to take part into the editing process.

**Fig. 6:**
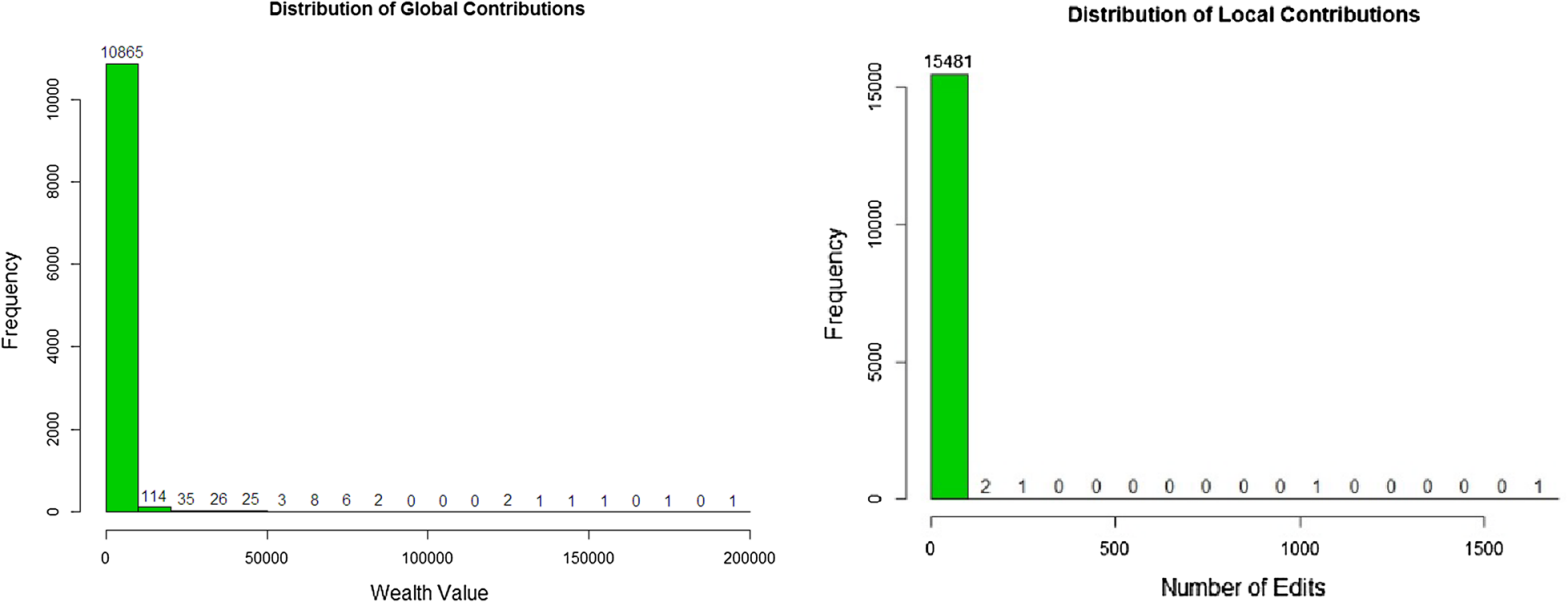
Distribution of users’ global and local contributions

One interesting part of critical mass theory is that collective action usually requires reaching critical mass—a small segment of the population that chooses to make big contributions to the collective action while the majority does little or nothing. These small segments that make large contributions to collective action are the critical mass for that the collective action. Note that, in the Baidu Encyclopedia community, each user’s number of edits in sub-community can be viewed as the local contribution while the wealth value can be viewed as the global contribution. In this way, the distributions of these users’ wealth value and number of edits can be used to describe this phenomenon as follows (Fig. 6):

The histograms of wealth value and number of edits indicates that 97.96 % contributors gather were concentrated at or near the low endpoint of the wealth value spectrum and 99.97 % of contributors were concentrated at or near the small endpoint by number of edits made. These results prove that most contributors (97.96 %/99.97 %) make little or no contribution to the articles while only small segment of contributors (2.04 %/0.03 %) make large contributions to these articles. Actually, most people simply edit the articles that they happen to be reading rather than make substantial contributions to these collective actions. Lih (2004) ever argued that the more an article is edited, the better it generally is. Similarly, the distribution of the number of edits per article is similar to the distribution the users’ contributions. In this way, as assessed by the number of edits, the heterogeneity of quality of articles was inferred.

## Conclusions

Peer production systems are a new mode of production, and they are increasingly influencing our lives. Online encyclopedia-based Wiki systems play an important role in the transmission and sharing of knowledge. In these virtual communities, large numbers of volunteers transmit and share their knowledge by editing articles. Any Internet user can potentially benefit from these articles. In this way, making and improving these articles can be viewed as public good. Baidu Encyclopedia and Wikipedia provide a simple and easily operate platform for these volunteers to promote collaboration. In this way, the Baidu Encyclopedia has also survived the start-up problem, which is challenging for some collective action.

Critical mass theory was here applied to Baidu Encyclopedia to explain the emergence and to predict the sustainability of the knowledge sharing action in virtual communities and to study the crucial factors by which collective action would emerges and becomes self-sustaining. These results show the following: (1) Users of the Baidu Encyclopedia have many different reasons for editing articles, such as enjoyment, altruism, reciprocity, and earning more points. (2) Users’ personal resources, which included a variety of career and educational backgrounds, were significantly different. Most contributors are well-educated and still involved in the educational field, often as students. Knowledge communities should encourage those who are educated or well-informed to edit and evaluate the articles. (3) The scoring rules used to evaluate the user’s contributions and assess each user’s reward and honor shows that the type of production function is accelerating function, but also the production function assessed by the number of contributions versus the number of edits over time also showed an accelerating function. These results were entirely consistent with the argument that a belief in furthering the collective good causes production function to accelerate. These results also indicate that an increasing number of users may take part into the collective workspace. (4) The distribution of users’ local and global contributions also indicated that in these communities, only a small segment of the population chose to make large contributions to the collective action while the majority did little or nothing.

Other statistical characteristics of articles, such as the number of edits undergone and the number of contributors involved can also be extracted. These results show that most articles in Baidu Encyclopedia are still in their initial stages. More articles need to be further modified.

In summary, the virtual community of the Baidu Encyclopedia can reduce the costs of creating and publishing information, which also reduces barrier to participation. The development of the community mainly involves on the persistent contributions of heterogeneous groups of users with many different motivations and ways of participating. Analyses show that Baidu Encyclopedia meets key conditions that the collective actions of knowledge contribution emerge and become self-sustaining. Increasing numbers of users then actively participate in discussions and creation of content on the Internet.

In addition, the insight can be obtained from current analyses for other similar existing communities: It is important to establish mechanisms that may attract different kinds of users, especially educated or well-informed users, and keep them involved. There is no denying that this study has some limitations. First, there may be other factors that can influence the collective action of knowledge sharing among virtual communities. Secondly, only career and level of education was used to explain the resource heterogeneity. Certainly, more factors needed to be evaluated to facilitate analysis and explain heterogeneity. It is currently difficult to directly assess the content production function. In this way, we not only explained the type of production function using the score rule, but also adopted the curve fit to assess the type of production function. However, more reasonable methods of assessing the type of production function are planned.
